# Environmental determinants influencing anthrax distribution in Queen Elizabeth Protected Area, Western Uganda

**DOI:** 10.1371/journal.pone.0237223

**Published:** 2020-08-18

**Authors:** Margaret Driciru, Innocent B. Rwego, Sood A. Ndimuligo, Dominic A. Travis, Elibariki R. Mwakapeje, Meggan Craft, Benon Asiimwe, Julio Alvarez, Samuel Ayebare, Katharine Pelican

**Affiliations:** 1 Queen Elizabeth National Park, Uganda Wildlife Authority, Kampala, Uganda; 2 Department of Biosecurity, Ecosystems and Veterinary Public Health, College of Veterinary Medicine, Animal Resources and Biosecurity, Makerere University, Kampala, Uganda; 3 Department of Veterinary Population Medicine, College of Veterinary Medicine, University of Minnesota, St. Paul, Minneapolis, United States of America; 4 Centre for Ecological and Evolutionary Synthesis, University of Oslo, Oslo, Norway; 5 Epidemiology and Diseases Control Section, Ministry of Health, Community Development, Gender, Elderly and Children, Dar es Salaam, Tanzania; 6 Department of Veterinary Medicine and Public Health, Sokoine University of Agriculture, Morogoro, Tanzania; 7 Department of Medical Microbiology, College of Health Sciences, Makerere University, Kampala, Uganda; 8 VISAVET Health Surveillance Center, Universidad Complutense, Madrid, Spain; 9 Departamento de Sanidad Animal, Facultad de Veterinaria, Universidad Complutense, Madrid, Spain; 10 Wildlife Conservation Society, Bronx, New York City, NY, United States of America; Spectrum Health, UNITED STATES

## Abstract

*Bacillus anthracis*, the bacteria that causes anthrax, a disease that primarily affects herbivorous animals, is a soil borne endospore-forming microbe. Environmental distribution of viable spores determines risky landscapes for herbivore exposure and subsequent anthrax outbreaks. Spore survival and longevity depends on suitable conditions in its environment. Anthrax is endemic in Queen Elizabeth Protected Area in western Uganda. Periodic historical outbreaks with significant wildlife losses date to 1950s, but *B*. *anthracis* ecological niche in the ecosystem is poorly understood. This study used the Maximum Entropy modeling algorithm method to predict suitable niche and environmental conditions that may support anthrax distribution and spore survival. Model inputs comprised 471 presence-only anthrax occurrence data from park management records of 1956–2010, and 11 predictor variables derived from the World Climatic and Africa Soil Grids online resources, selected considering the ecology of anthrax. The findings revealed predicted suitable niche favoring survival and distribution of anthrax spores as a narrow-restricted corridor within the study area, defined by hot-dry climatic conditions with alkaline soils rich in potassium and calcium. A mean test AUC of 0.94 and predicted probability of 0.93 for anthrax presence were registered. The five most important predictor variables that accounted for 93.8% of model variability were annual precipitation (70.1%), exchangeable potassium (12.6%), annual mean temperature (4.3%), soil pH (3.7%) and calcium (3.1%). The predicted suitable soil properties likely originate from existing sedimentary calcareous gypsum rocks. This has implications for long-term presence of *B*. *anthracis* spores and might explain the long history of anthrax experienced in the area. However, occurrence of suitable niche as a restricted hot zone offers opportunities for targeted anthrax surveillance, response and establishment of monitoring strategies in QEPA.

## Introduction

*Bacillus anthracis*, the bacteria that causes anthrax, a disease that primarily affects herbivorous animals, is a soil borne endospore-forming microbe [1–3]. *B*. *anthracis* infectious cycles comprise: - 1) an infective vegetative form that circulates inside a susceptible host in which the bacteria elicits the formation of toxic complexes that cause host death; and 2) a dormant spore form that circulates in the soil, but sporulates from vegetative bacteria shed in haemorrhagic exudates upon host death [2–4]. Soils contaminated with terminally haemorrhaged blood and other body exudates after the death of an infected host are seeded with high levels of *B*. *anthracis* spores [5], that have the potential, upon ingestion to establish an animal-soil-animal cycle [3] that forms a risky interface for susceptible herbivores [6–8]. Distribution of viable *B*. *anthracis* spores in the environment determines exposure risks for grazing herbivores and subsequent anthrax outbreaks [2, 9]. In its sporulated form, *B*. *anthracis* spores become dormant and very resistant to harsh environmental conditions such as heat, dehydration, pH, desiccation, chemicals, irradiation, and this state plays a central role in maintenance of anthrax in the ecosystem [1, 10, 11]. Viability and longevity of spores in the soil is reported to be influenced by levels of soil calcium, moisture, and alkalinity; hot-dry weather, mean annual temperatures, annual precipitation; elevation, and vegetation types [3, 9, 12]. In particular, calcium (Ca2+) forms an integral component of the core region of bacterial spores where it plays an important role in stabilizing spores during periods of dormancy and maintains viability of spores in the soil for extended lengths of time [4]. A common soil-dwelling amoeba, *Acanthamoeba castellanii*, has also been shown to contribute to persistence of *B*. *anthracis* spores in natural environments by enhancing spore germination, multiplication and amplification processes [13].

Globally, it is estimated that 1.1 billion livestock and 1.83 billion people live at a high-risk interphase with anthrax spores [6]. This risk is higher for grazing herbivores exposed on contaminated pasture, soil and water than it is for humans [2, 3, 8, 14].

Knowledge of exposure risks to infectious agents, pathways for their transmission and pathogenesis are fundamentals for disease control and prevention [2, 15–17], ecosystem health management and species conservation in wildlife populations. Anthrax has a long-standing history in Queen Elizabeth Protected Area (QEPA) in Western Uganda, with a well-established animal-soil-animal cycle, resulting in sporadic and large-scale periodic outbreaks [18, 19]. Despite this history, environmental drivers potentiating outbreaks in the ecosystem have not been assessed, and outbreaks continue to cause severe losses of key wildlife species and poise challenges for conservation, tourism and public health.

Ecological niche models (ENM) are tools that have greatly improved understanding of suitable environments that support species survival for ecological studies [20–23], and have been extensively used for anthrax ecology studies [6, 9, 24, 25]. This study aimed to estimate the suitable landscape and environmental predictors that support persistent survival of *B*. *anthracis* spores in the study area.

## Materials and methods

### Study area

This study was conducted in Queen Elizabeth Protected Area (QEPA), a mixed savannah-woodland-forest wildlife ecosystem in South Western Uganda (S1 Fig in Driciru *et al* [30]). The QEPA reserve lies at the floor of the Great East African Rift Valley system, and is closely surrounded by agricultural land and cattle corridors. The geological formations and associated volcanicity of the Rift Valley have resulted in alkaline soils rich in volcanic ash with high levels of phosphorus, and calcium [26]. Climatic conditions seasonally cycle between warm-wet (March to May and August to November); and hot-dry months (December to February, and June to July) [27–29]. These soil, climatic and biological conditions have been hypothesized as conducive for eliciting anthrax outbreaks and sustaining *B*. *anthracis* spores in soils of the study area [30], and influenced selection of predictor variables.

The study extent for modeling encompassed QEPA and the surrounding ecosystem and was created using the layer features for polygon shapefiles tool in QGIS (Free Software Foundation, Inc., 51 Franklin Street, Boston, USA). Rasterized layers of predictor variables and bias layer used were projected to a common coordinate system (WGS84), the layers were masked, clipped to the study area extent using the raster clipper tool and saved in ASCII (.asc) format for modeling.

### Data collection

#### Response variables

A dataset of 471 geo-referenced presence-only anthrax occurrence records from both clinical and laboratory confirmed outbreak cases were used. Of these, 188 records were from 13 historical outbreaks that occurred in QEPA, between 1956 to 1979 involving, 601 wildlife mortalities, from hippopotamus (90.1%), Cape buffalo (7.5%); and Uganda kob, waterbuck, elephant, warthog, and lion (2.4%). The remaining 383 records were from two recent epidemics that occurred in QEPA between 2004 and 2010 involving 536 wildlife mortalities, with similar species distribution patterns: hippos (82.8%), cape buffalo (13.8%) and others (3.8%) [18, 30]. The referenced historical data records were mined from archives (S1 Fig) of: - 1) telegram communications; 2) diary and journal books of the then wildlife managers/wardens; 3) field epidemiologist’s disease incidence reporting, diagnosis and outbreak investigation reports; 4) park management quarterly and annual reports; and 5) annual reports of the Animal Health Research Centre (AHRC), Department of Veterinary Services and Animal Industry, Entebbe, Uganda [31]. As a standard practice during disease outbreak investigations, diagnostic samples are often taken from a few affected animals to confirm disease etiology. Based on the clinical or pathological signs in confirmed or related cases, a case definition is developed and signs common to all affected animals is then used for enumerating cases used for epidemic analysis [15, 16]. In this study, historical mortality records considered as confirmed cases were from outbreaks for which laboratory diagnostic records were available (S1 Fig), and suspected cases were those that described pathognomonic clinical sign(s) based on the standard clinical case definition for anthrax in animals [2, 32], such as bloating and oozing of un-clotted dark coloured blood from natural body orifices. The referenced diagnostic records used bacteriological staining techniques (S1 Fig) [31] and PCR for more recent outbreaks [18]. Included case records also contained information on geographic location, animal species affected and case numbers. For cases with missing coordinates, GPS waypoints were marked during the study by tracing back location information provided in the original record.

#### Predictor variables

A total of 44 environmental and bioclimatic predictor variables commonly used in ecological and species distribution modeling [25, 33] were considered for prediction of the suitable niche for anthrax in the study area (Table 1). Selection criteria considered variable properties that influence sporulation, survival, germination, or dissemination of *B*. *anthracis* spores in the soil [2–4, 12]. Bioclimatic variables used were derived from monthly temperature and rainfall values from the World Climate data (http://worldclim.org/version2) [20]. This data set contains 19 variables, comprised of 11 temperature (Bio1 –Bio11) and 8 precipitation covariates (Bio 12 –Bio 19), (Table 1). Data used are averages for the period 1970–2000 (30 years), and values are measured at a 1 km^2^ (30 seconds) spatial resolution.

**Table 1:** Bioclimatic predictor variables used for modeling suitable environmental conditions influencing anthrax distribution in QEPA.

S/N	Variable	Variable definition
		***Temperature variables***
1.	Bio1	Annual Mean Temperature
2.	Bio2	Annual Mean Diurnal Range (Mean of monthly (max temp—min temp)
3.	Bio3	Isothermality (BIO2/BIO7) (* 100)
4.	Bio4	Temperature Seasonality (standard deviation *100)
5.	Bio5	Max Temperature of Warmest Month
6.	Bio6	Min Temperature of Coldest Month
7.	Bio7	Temperature Annual Range (BIO5-BIO6)
8.	Bio8	Mean Temperature of Wettest Quarter
9.	Bio9	Mean Temperature of Driest Quarter
10.	Bio10	Mean Temperature of Warmest Quarter
11.	Bio11	Mean Temperature of Coldest Quarter
		***Rainfall variables***
12.	Bio12	Annual Precipitation
13.	Bio13	Precipitation of Wettest Month
14.	Bio14	Precipitation of Driest Month
15.	Bio15	Precipitation Seasonality (Coefficient of Variation)
16.	Bio16	Precipitation of Wettest Quarter
17.	Bio17	Precipitation of Driest Quarter
18.	Bio18	Precipitation of Warmest Quarter
19.	Bio19	Precipitation of Coldest Quarter

*Source*: The bioclimatic data series provides GIS continuous raster surfaces that represent multiple temporal and spatial resolutions. Climate normal are 30-year monthly averaged temperature and precipitation data between 1971 and 2000 (30 years inclusive); maximum and minimum temperatures for monthly data reflect the monthly means of daily maximum temperatures and monthly means of daily minimum temperatures [33].

Soil variables were derived from the Africa SoilGrids online resources (ISRIC), predicted using two point datasets for Africa soil profile datasets and Africa Soil Information Services (AfSIS) Sentinel Site database (https://www.isric.org/projects/soil-property-maps-africa-250-m-resolution). Fifteen soil variables selected (Table 2) included soil type, pH, exchangeable calcium (Ca^2+^), potassium (K^+^), sodium (Na^+^), magnesium (Mg^2+^); extractable and total phosphorous (P), nitrogen, and soil organic carbon (SOC) [34]. These data were selected at soil depths of 0–20 cm and 20–50 cm, where the nutrients are considered available for uptake by plants and soil micro-organisms; and the data layers used were at a spatial resolution of 250 m. Variables like calcium and pH are known to maintain spore viability in the soil and influence germination processes [4, 12]. Other variables used included Digital Elevation Model (DEM), slope, aspect, drainage (distance to small and seasonal rivers), lithology, cloud cover, landcover types and frequency of fires. A 90-m DEM was obtained from the United States Geological survey [35]. Drainage was derived by calculating Euclidian distance from rivers; slope and aspect were derived from the DEM using spatial analysis tools in ArcGIS. The Lithology layer represents the key geological parent materials [36]. Cloud cover layers used were derived from MODIS Surface reflectance data computed by Guy Picton Phillips [37]. Land cover and fire frequency layers were provided by Wildlife Conservation Society, Uganda program and mapped from Lands at imagery [38].

**Table 2:** Soil properties and geo-physical variables used for modeling suitable environmental conditions influencing anthrax distribution in QEPA.

S/N	Variable	Variable Definition
1.	soil_cl	Soil types
2.	calcium_0_20cm	Exchangeable calcium at soil depth of 0_20cm
3.	calcium_20_50cm	Exchangeable calcium at soil depth of 20_50cm
4.	magnesium_0_20cm	Exchangeable magnesium at soil depths of 0_20cm
5.	nitrogen_0_20cm	Total nitrogen measured at soil depth of 0_20cm
6.	nitrogen_20_50cm	Total nitrogen measured at soil depth of 20_50cm
7.	ph_0_5cm	Soil pH at depth of 0-5cm
8.	ph_5_15cm	Soil pH at depth of 5-15cm
9.	ph_60_100cm	Soil pH at depth of 60-100cm
10.	phospho_extract_0_30cm	Extractable phosphorus measure at soil depth of 0_30cm
11.	phospho_total_0_30cm	Total phosphorus measure at soil depth of 0_30cm
12.	potasium_0_20cm	Exchangeable Potassium measured at soil depth of 0-20cm
13.	sodium_0_20cm	Exchangeable Sodium measured at soil depth of 0-20cm
14.	soil_carbon_0_5cm	Soil organic carbon measured at depths of 0_5cm
15.	soil_carbon_5_15cm	Soil organic carbon measured at depths of 5_15cm
16.	Aspect	Direction of the slope
17.	cloud_max	Maximum cloud cover
18.	cloud_mean	Mean cloud cover
19.	DEM	Digital Elevation Model (Altitude)
20.	dist_rivers_small_seasnl	Distance from small and seasonal rivers (joined layer)
21.	fires_2004_2017	Fire frequencies per pixel
22.	ker_hippo_2006_18	Mean kennel density of hippos (2006–2018)
23.	Landcvr	Land cover
24.	Slope	Slope
25.	Lithology	Parental rock material

### Model development

Collinearity is a concern in regression models, as highly correlated predictor variables cannot independently predict the value of the dependent variable, since they will be explaining the same variance in the dependent variable, which in turn reduces their statistical significance. To minimize the effect of multi-collinearity and model over fitting, highly correlated predictor variables (n = 44) were discriminated using the pair wise Pearson correlation statistics in ENMTOOLs [24, 39, 40] at cut off (r ≥ 0.75) (S1 Table). At r ≤0.75, variables were selected for inclusion for modelling, otherwise dropped (r ≥ 0.75) and only one of the pair included. The resulting non correlated variables (n = 23) were further discriminated using Maxent model jackknifing procedures that ranks variables according to their percent contribution towards model development. A total of 11 predictor variables (Table 3), and 122 anthrax occurrence localities from all recorded species randomly selected by the model from a total of 471 from all representative grid cells were used for building the final models. Hippos being the most susceptible wildlife species that suffer significant mortality due to anthrax at the study area, contributed > 80% of occurrence localities used for the ENM modelling in this study. To assess the effect of hippos as a confounding factor in determining suitable anthrax niche, an independent model was built without hippo, but using 40 occurrence localities from buffalo only (partitioned 3:1, for model building and calibration).

**Table 3:** Final predictor variables used in modeling suitable environmental conditions supporting anthrax distribution and spore survival in QEPA.

S/N	Variable	Unit of measurement
1.	Annual precipitation	Millimeters (mm)
2.	Precipitation of Coldest Quarter	Millimeters (mm)
3.	Annual Mean Temperature	^0^C
4.	Exchangeable potassium (0_20cm)	cmolc/kg
5.	Exchangeable calcium (20_50cm)	cmolc/kg
6.	Exchangeable Sodium (0_20cm)	cmolc/kg
7.	Soil pH (5_15cm)	NA
8.	Soil Organic carbon (5_15cm)	g/kg
9.	Soil types	NA
10.	Land cover	NA
11.	Fire frequencies/burn scars	NA

The proportion of total response records input for Maxent modelling that are modelled depends on the resolution of predictor variables, in our study, grid cells used had a resolution of one km and 26% of response variables input were used. Maxent assigns all records found in the same grid cell one value as it requires only one location to infer suitable conditions per cell, hence the rest of the records are considered duplicates and automatically removed. This is aimed at minimizing model over fitting due to sampling biases likely to be encountered during data collection [22], but can potentially underrepresent areas experiencing high disease incidence. We used a bias layer rasterized from spatial records comprising of all occurrence points to essentially represent areas with comparatively higher incidences.

Occurrence records used were partitioned into a ratio of 3:1, a percentage of 75% (n = 92) were used for building and calibrating the model, and 25% (n = 30) for testing the predictive power of the models following Pearson’s guidelines [21, 23, 41].

The maximum entropy (Maxent version 3.4.1) ecological niche modeling algorithm method was used to predict suitable niche that supports the survival and geographic distribution of *B*. *anthracis* in the study area following guidelines of Phillips *et al* [21, 22]. Maxent ENM tools have a tuning method that uses presence-only data and this was appropriate for the study data type, but other modelling tools like the Logistic Regression model require binary data containing species presence-absence records as dependent variables [22]. Default auto features recommended as optimal values by model developers were mostly used to run the model, but with a beta multiplier of 8, hinge product linear threshold quadratic feature types and logistic output format. Model fitting was assessed using 100 replicate model runs (bootstraps), at a default maximum number of 1,000 iterations. A total of 6,795 background and presence points randomly generated from the covariate space were used to determine the Maxent distribution. Maxent utilizes associations between environmental variables and known species occurrence localities to predict potentially suitable environmental conditions within which a species can survive [23]. True species distribution is presented as a probability distribution over a set of pixelated sampled sites of the 1.0 km^2^ grid cells in the study area. The model output value returns a predicted habitat suitability reported as a logistic score (0–1) which is dependent on the feature or environmental predictor variables at the site [22, 39]. During each iteration, every variable is omitted in turn or used in isolation or in combination with all others and a model is built for the corresponding variable. Variable contribution towards model development was assessed using the percent relative increase in the regularized training gain, and the importance of every variable towards the predictive power of the model assessed using Jackknife statistical techniques. Response curves built during the modeling process were used to evaluate how varying levels of each environmental variable affected the ability of the model to predict suitable areas for survival of *B*. *anthracis* in the study environment by measuring the change in the predictive logistic scores.

### Model evaluation

Model accuracy and predictive performance was assessed using a threshold-independent evaluation technique, derived using the Area Under the Receiver Operating Characteristic Curve (AUC) statistics and a logistic output format [23, 42]. For presence-only modeling using Maxent, the AUC measures the probability that a randomly chosen presence site will be ranked higher than a randomly chosen background site [22], and this in essence implies a better discriminative power between presence versus absence sites [43]. AUC values range from 0.5 for models that are no better than random chance to 1.0 for models with perfect predictive ability. AUC values of 0.6–0.7 are classified as poor, 0.7–0.8 as average, 0.8–0.9 good, and 0.9–1.0 as excellent [23, 25]. This implies that for AUC values ≥ 0.80, the model has a higher than random chance that a randomly selected presence site would contain a higher predictive value than a randomly selected background site [43].

### Ethical statements for the research project

This study forms the second component of a bigger research project on ecology of anthrax at the study site. Research approvals for the bigger project, as outlined in [30], were obtained from Uganda National Council for Science and Technology (UNCST) (Ref: NS 418); and Uganda Wildlife Authority (UWA) (Ref: UWA/TDO/33/02) for research in Protected Areas involving wildlife. Ethical approvals, research protocols, tools and bio-safety considerations that have been used for other components of the research project were reviewed and approved by two Institutional Review Boards (IRB) and Research Ethics Committees from College of Health Sciences, School of Medicine (#REC REF 2013–084); and College of Veterinary Medicine, Animal Resources and Biosecurity (COVAB), School of Veterinary Medicine (VAB/BRC/14/101) of Makerere University, Kampala, Uganda.

## Results

### Model performance

**Fig 1:**
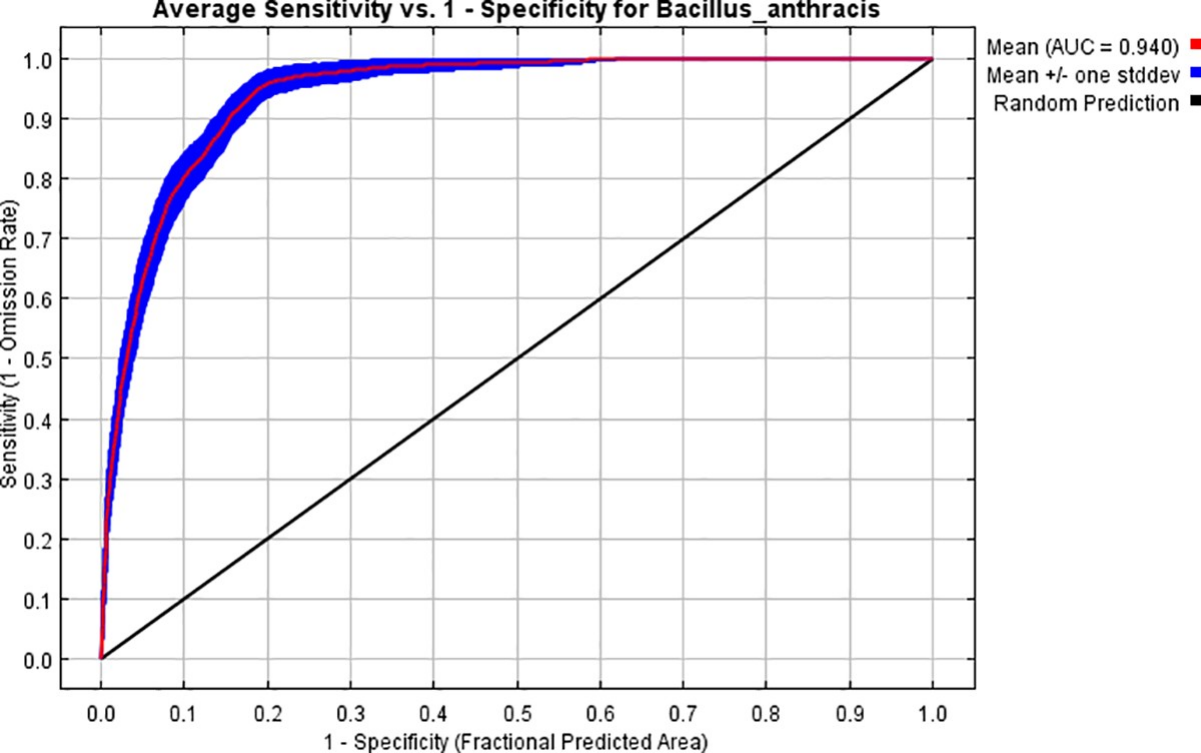
Mean AUC for 100 model replicate runs for predicting occurrence of *B*. *anthracis* at QEPA. AUC stands for “Area under the Receiver Operating Characteristic (ROC) Curve”. Specificity is defined using predicted area, rather than commission.

Eleven (n = 11) environmental predictor variables screened from a total of 44 (Table 3) were determined to be non-correlated (r ≥ 0.75) and of good fit, these were used to develop the final Maxent species survival and distribution model for *B*. *anthracis* in QEPA. The mean test AUC score was 0.936±0.015 (95% Confidence Interval—CI), and training AUC was 0.94±0.008 for the 100 replicate models run (Fig 1). A mean test omission rate of 5.2% and training omission rate of 8.9% were achieved, implying that 94.8% of test points used for validating the model were correctly predicted. The AUC values were significantly (p < 0.0001) higher than Maxent’s random prediction baseline value of 0.5 for models that are no better than random and therefore demonstrate good model accuracy. This implies performance of the model on validation (test) data (n = 30) for accurately predicting presence locations was excellent.

#### Variable contribution

Of the 11 environmental predictor variables modeled, five contributed most towards building the *B*. *anthracis* species survival model for QEPA. Of these, annual precipitation (*bio12_cl*) made the greatest relative percent contribution (70.1%) followed by exchangeable potassium ions measured at soil depth of 0-20cm (12.6%), annual mean temperature (bio1_cl), 4.3%, soil pH at 5-15cm (3.7%), and exchangeable calcium at 20-50cm (3.1%) (Table 4).

**Table 4:** Values measuring importance of variables in contributing to development and predictive power of the Maxent model for predicting suitable conditions for *B*. *anthracis* survival in QEPA.

S/N	Variable ID	Variable definition	Percent (%) contribution	Permutation importance	Jacknife AUC Score	Predicted suitability score
1.	Bio12_cl	Annual precipitation	70.1	83.1	0.93	0.90
2.	Potassium_0_20cm	Exchangeable potassium at 0-20cm soil depth	12.6	1.9	0.76	0.95
3.	Bio1_cl	Annual Mean Temperature	4.3	11.4	0.78	0.67
4.	pH_5_15cm	Soil pH at 5-15cm soil depth	3.7	0.2	0.81	0.90
5.	Calcium 20_50cm	Exchangeable calcium at 20–50cm	3.1	1.0	0.79	0.91
6.	Landcvr	Land cover	2.0	0.6	0.72	NA
8.	Bio19_cl	Precipitation of Coldest Quarter	1.3	0.4	0.87	0.61
7.	Fires_2004_2017	Fire frequencies/burn scars	1.3	0.4	0.72	0.80
9.	Soil_cl	Soil types	1.2	0.7	0.76	NA
10.	Soil_carbon_5_15cm	Organic carbon at 5_15cm depth	0.2	0.3	0.72	0.70
11.	sodium_0_20cm	Exchangeable Sodium at 0-20cm	0.1	0	0.79	0.60

AUC thresholds of 0.6–0.7 are classified as poor, 0.7–0.8 as average, 0.8–0.9 good, 0.9–1.0 as excellent [23, 25]. The jackknife test AUC and suitability scores assess individual variable importance to the prediction and are measured for the individual variables in isolation of the others.

**Fig 2:**
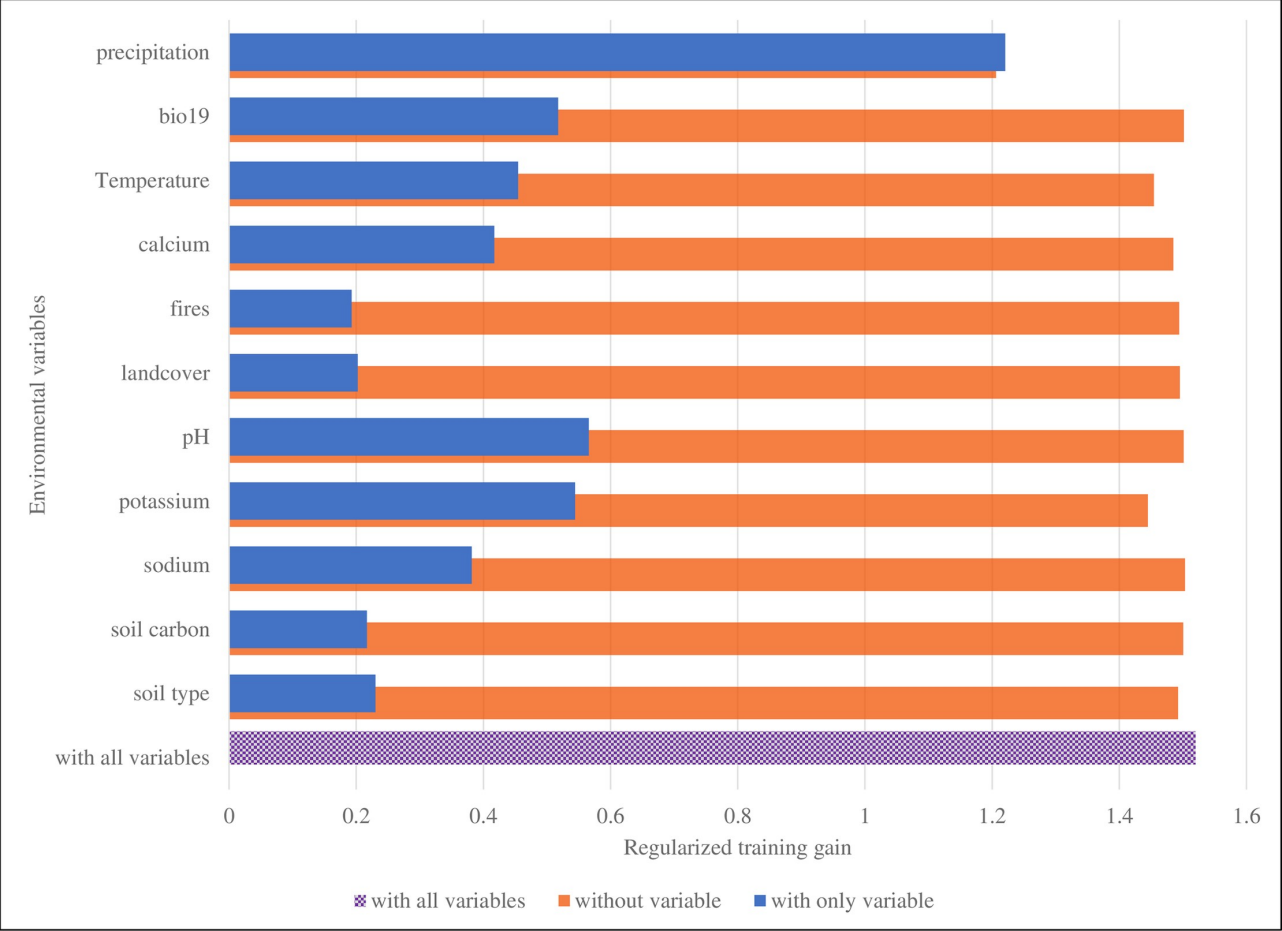
Jacknife test assessing variable importance using regularized training gain for building the *B*. *anthracis* species survival model. Variable of greatest importance for building the model was bio12_cl. When used in isolation, it contributed the highest gain (1.2) and when omitted, decreased the gain the most.

**Fig 3:**
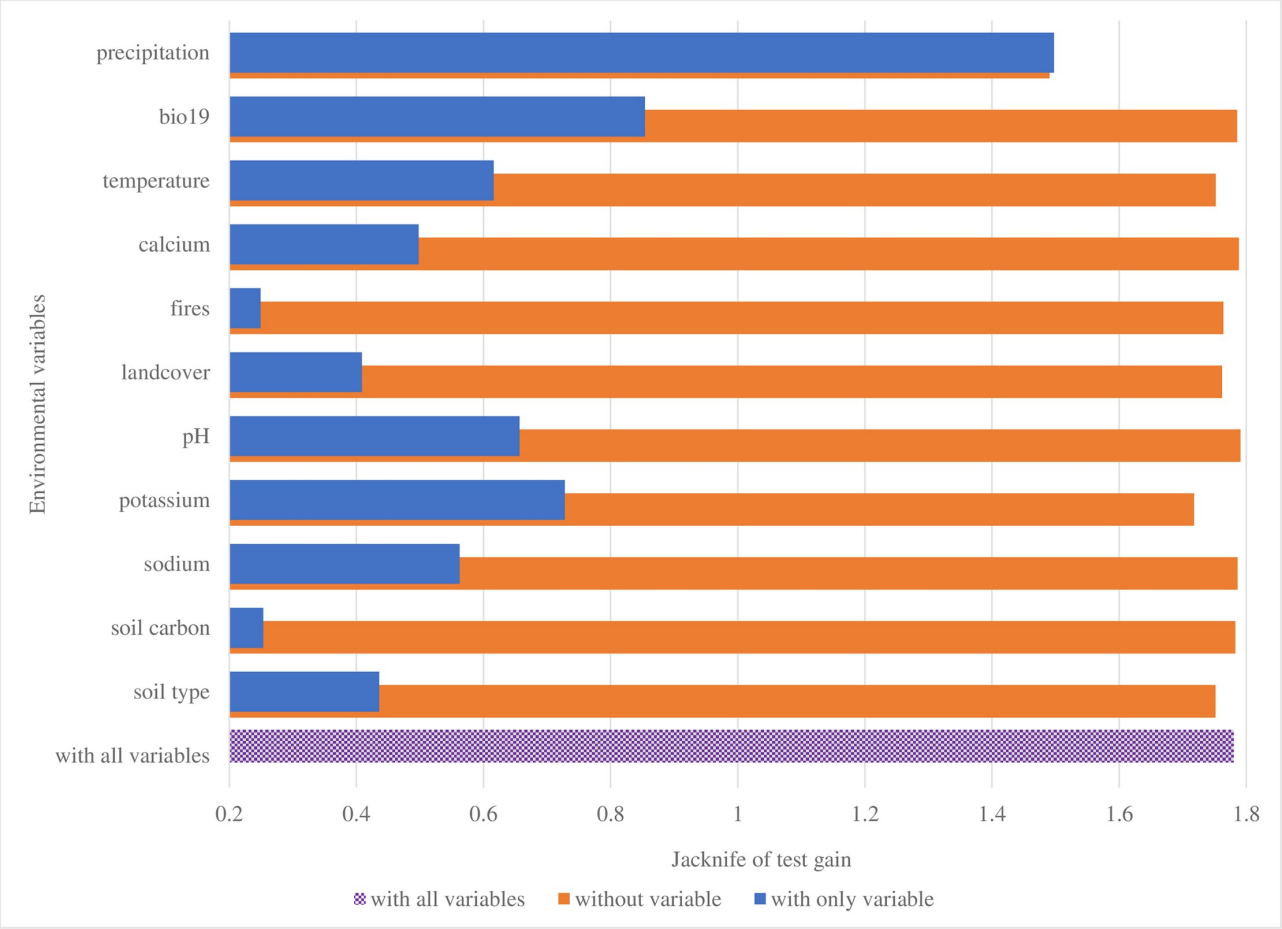
Jacknife test for assessing variable importance using test gain for validating the *B*. *anthracis* species survival model. Variable of greatest importance for validating the B. anthracis survival model was bio12_cl. When used in isolation, it contributed the highest gain (1.5) when omitted, decreased the gain the most.

**Fig 4:**
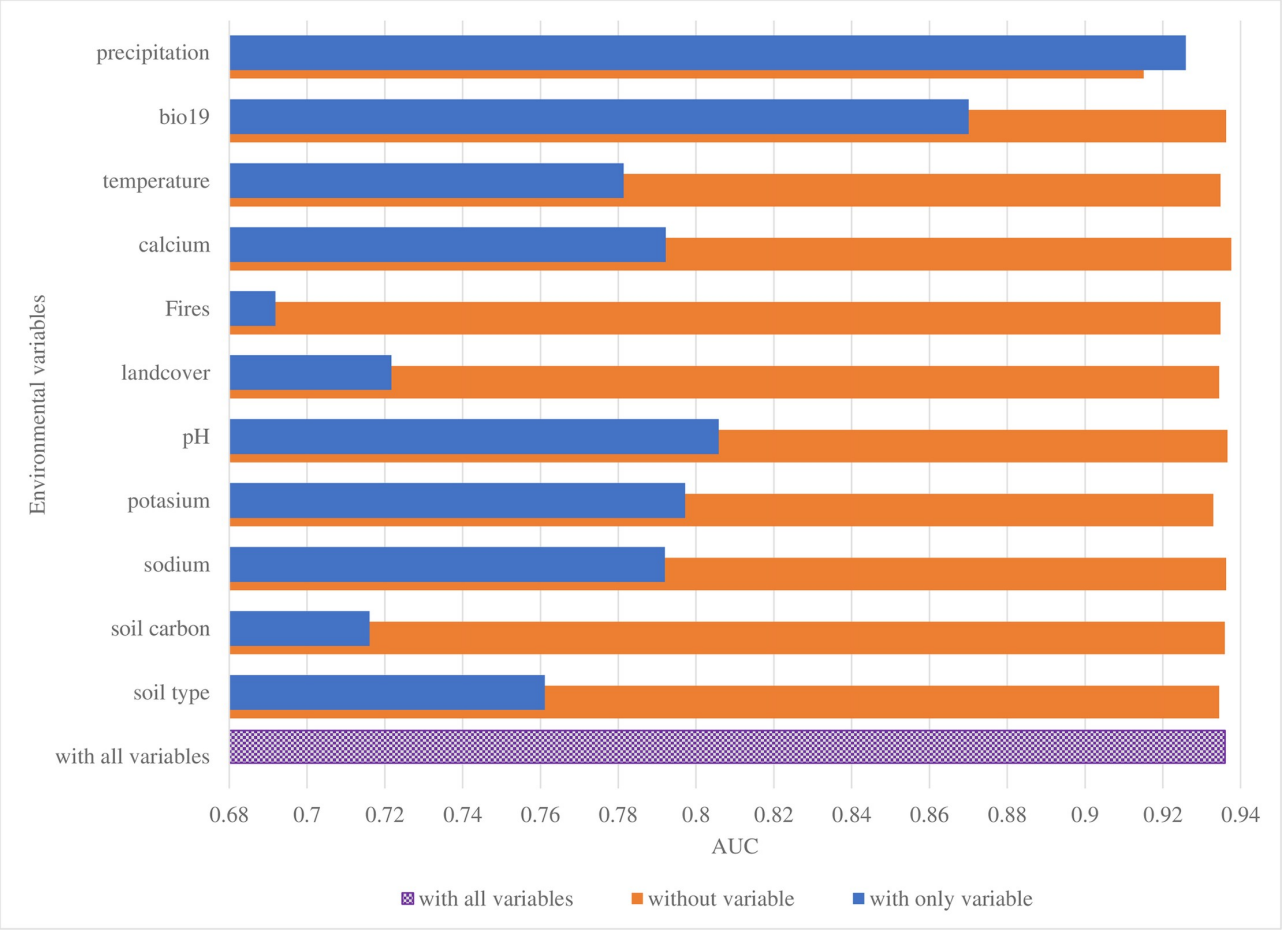
Jacknife test for assessing variable importance using Area under the receiver operating curve (AUC) on test data. Variable bio12_cl by itself registered the highest test AUC (0.925) for measuring model accuracy. All variables with AUC ≥0.75 had significant contribution to the model. AUC thresholds of 0.6–0.7 are classified as poor, 0.7–0.8 as average, 0.8–0.9 good, 0.9–1.0 as excellent [23, 25].

Jackknife tests helped to assess which particular variables were most important in the model, and results show that omitting each of the above five variables in turn or using each in isolation during model development and validation significantly affected the regularized training gain, AUC and test gain of the model (Figs 2–4). Annual precipitation was the most important variable (AUC 0.93) for the *B*. *anthracis* species survival model built for QEPA (Table 4). When used in isolation, this variable significantly increased both the test and regularized training gains for the model and omitting it significantly decreased the gain (Figs 2–4). This suggests that the variable contained the most useful information which was not present in the other variables. The second important variable that registered a good jackknife of test AUC score was soil pH (0.81), followed by calcium (0.79), annual mean temperature (0.78) and potassium (0.76).

### Predicted habitat suitability for *B*. *anthracis* spore survival

**Fig 5:**
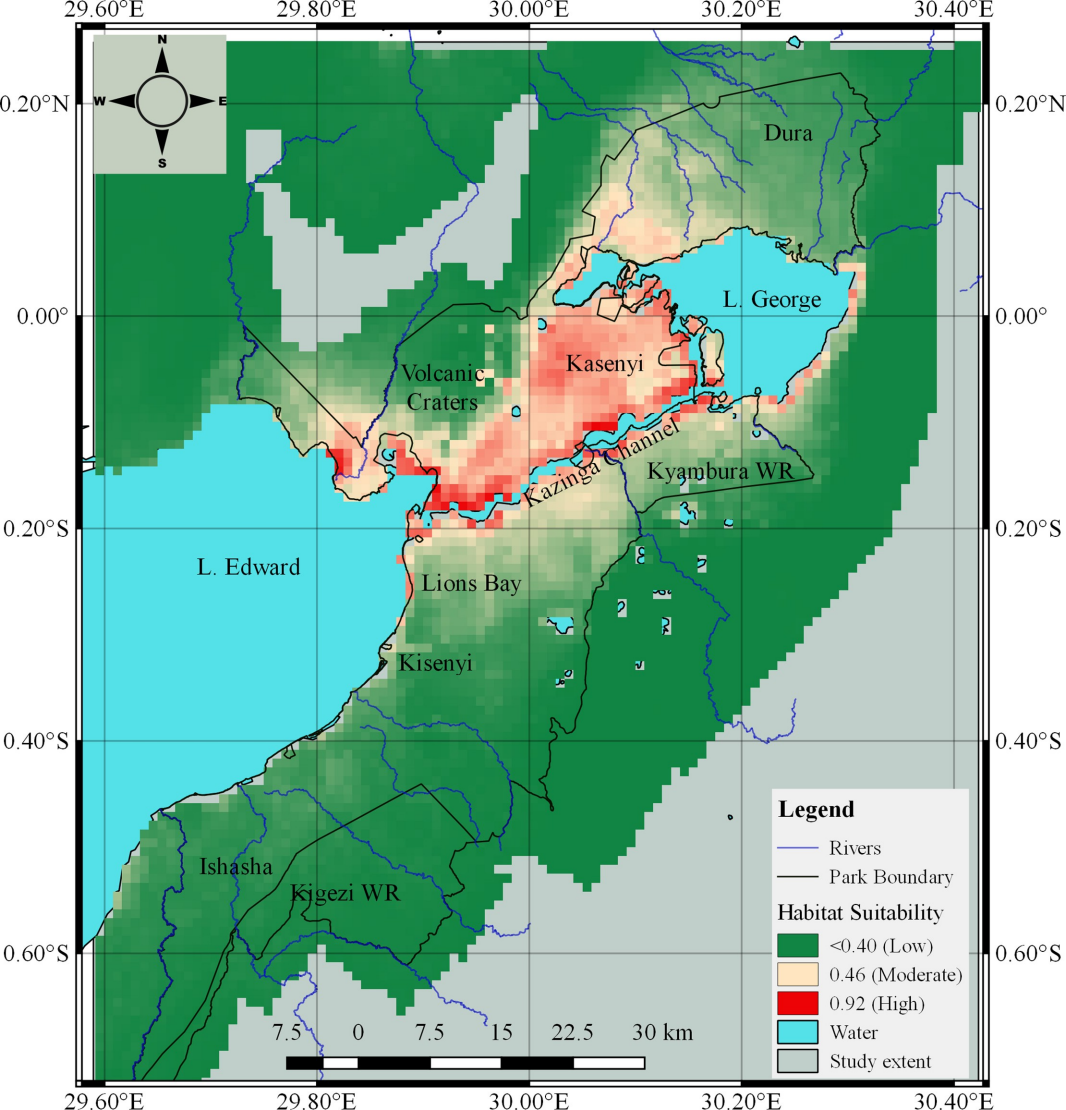
Predicted probability map showing habitats with suitable environmental conditions favouring *Bacillus anthracis* distribution in QEPA ecosystem. Red colour (0.927) shows areas with the highest probability of suitable conditions for anthrax occurrence, and green (<0.4) indicates areas predicted as least suitable sites. Predictor variables used for modelling obtained from WorldClim (http://worldclim.org/version2), and ISRIC online resources (https://www.isric.org/projects/soil-property-maps-africa-250-m-resolution), with kind permission of Dr. Stephen Fick, geo-spatial data scientist and Niels Batjes, Senior Soil Scientist and Coordinator of the World Data center for soils at ISRIC–World Soil Information.

The highest overall predicted probability score for anthrax occurrence at the study area was 0.927 (92.7%), range 0.695–0.927 (69.5%– 92.7%), and non-suitable sites had a score below 23.2% (Fig 5). Spatially, the potential suitable habitat for *B*. *anthracis* occurrence covered a narrow corridor bearing a north-east to south-east ward direction, spanning from just above Lake George downwards to L. Edward, moving along shorelines of key water bodies and mostly covering areas within the National park. The open savannah plains of Kasenyi, northwest of Kazinga channel up to Katwe bay were most suitable for spore survival and anthrax occurrence. The southern parts of the study area in Ishasha, the massive volcanic explosion craters northeast of Katwe, the Immaramagambo forest and areas falling far off the park boundary were predicted least suitable for survival of anthrax spores (Fig 5).

A model built using buffalo occurrence localities without hippos assessed the confounding effect of hippos in influencing the suitable area for anthrax distribution at the study area, the results were not significantly different (S2 Fig) from the main model that was built using occurrence localities inclusive of hippos (Fig 5). The narrow southwest-northeast corridor spanning from north of L. Edward to north of L. George and the associated shorelines remained outstanding potential hot zones for persistence of anthrax in the study ecosystem.

Marginal response curves helped to evaluate how the predicted probability (habitat suitability) for presence of anthrax changed as each environmental variable was varied, keeping all other environmental variables at their average sample value.

**Fig 6:**
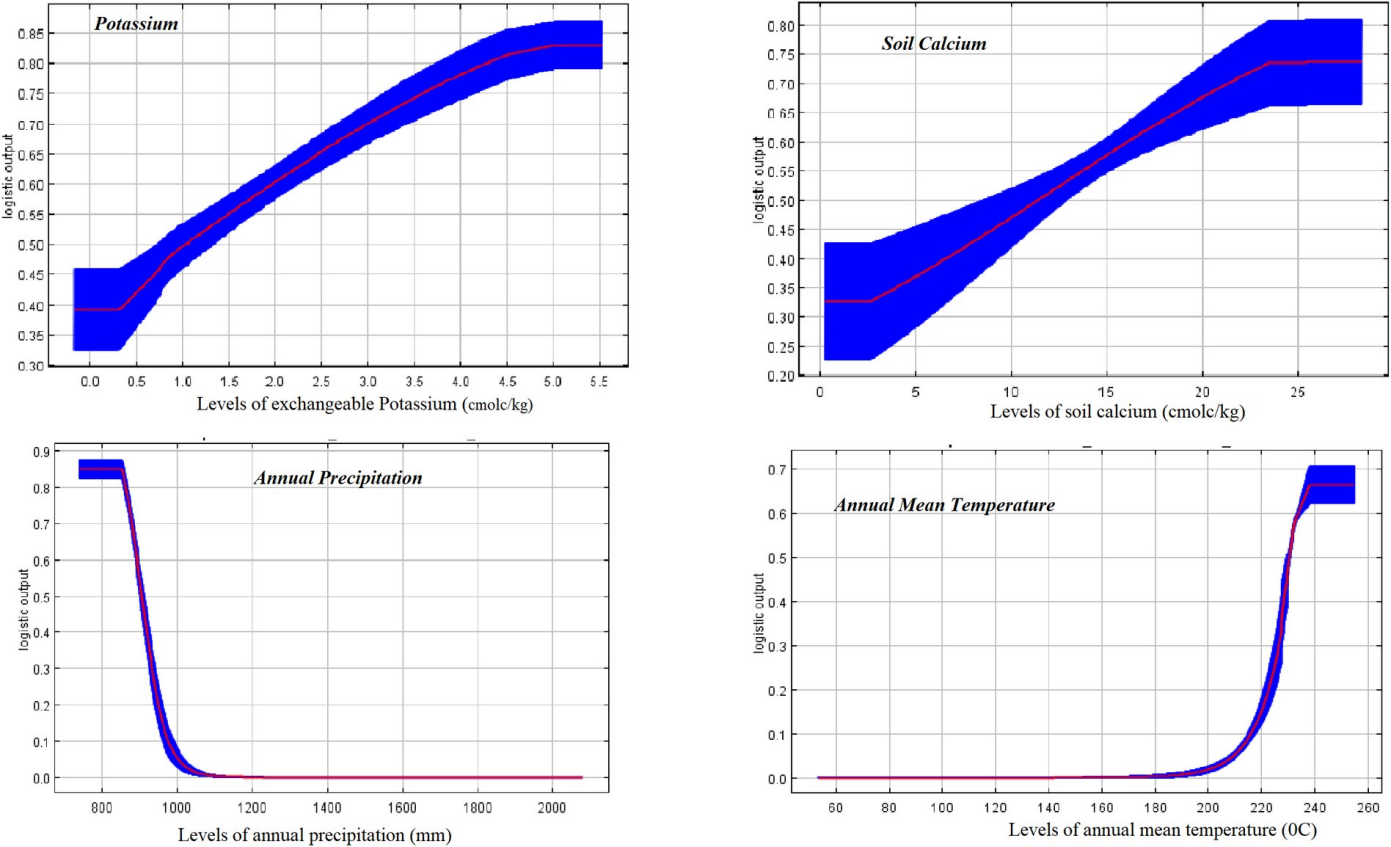
Marginal response curves showing how predicted suitability scores for anthrax presence changed with varying levels of annual precipitation, exchangeable potassium, soil calcium ions and annual mean temperature. Probability for anthrax spore survival increases with increasing levels of potassium, soil calcium ions and mean annual temperatures (Bio1_cl2). Probability for anthrax spore survival is however highest at lowest rainfall (Bio12_cl) amounts and drops drastically as precipitation levels increase. Values for annual mean temperature covariates are divisible by 10 to derive actual temperature value (http://worldclim.org/version2).

**Fig 7:**
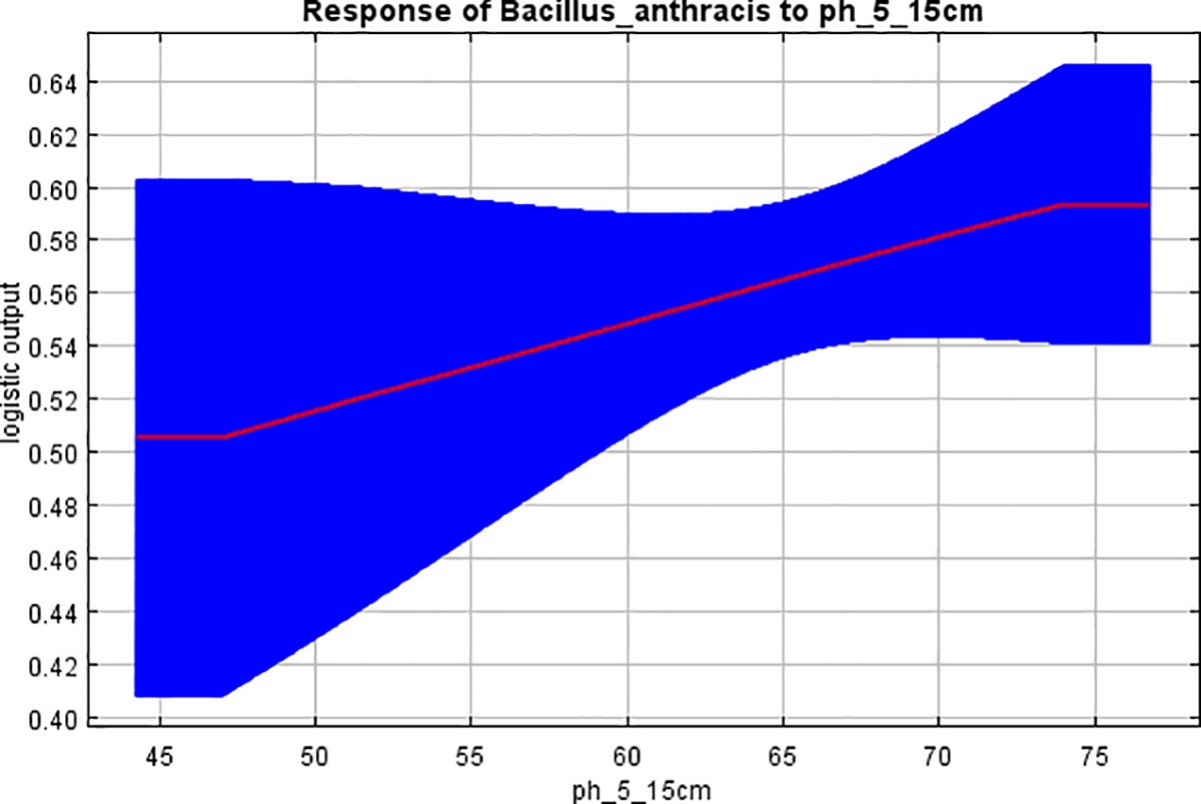
Marginal response curve showing how the predicted suitability for anthrax presence changed with varying levels of soil pH. Probability for anthrax spore survival increases with increasing levels of soil pH. Covariate values for pH are divisible by 10 to derive actual pH value.

Changing levels of the 5 most important variables to their optimal values significantly improved the marginal response and predictive performance of the model to predict presence of anthrax. Scores for the predicted probability of suitable conditions significantly improved with increasing levels of potassium (0.83 ± 0.03 Std), calcium (0.74 ± 0.08 Std), and pH (0.59 ± 0.05 Std) (Figs 6 & 7). Inverse levels of these variables resulted in lower marginal response and reduced predictive performance for the model. For annual precipitation, the probability scores were highest (0.85 ±0.03 Std) at the lowest levels of precipitation (≤ 825 mm) and drastically dropped with increased precipitation levels exceeding 1,000 mm (Fig 6). Response curves for models built using each corresponding variable in isolation consistently showed similar trends like those for marginal curves but yielded much higher suitability scores, ranging from 0.95 for potassium; 0.91 for calcium; 0.90 for pH and precipitation; and 0.67 for mean annual temperature (Table 4 & Fig 6).

Optimal values for variables predicting suitability were: potassium ≥5.044 cmolc/kg; calcium ≥25.9 cmolc/kg; pH ≥ 7.4; rainfall ≤ 852mm; and temperature ≥23.8°C respectively (Table 5).

**Table 5:** Optimal levels of environmental variables that most predicted suitable ecological niche for distribution and survival of anthrax spores at QEPA.

S/N	Variable	Predicted probability of anthrax occurrence		
		Low (≤0.0001)	Medium (0.232–0.464)	High (optimal) (0.695–0.927)
1.	Annual precipitation (mm)	1690–1970	1331–1411	≤ 852
2.	Exchangeable potassium at 0-20cm soil depth (cmolc/kg)	≤0.25	1.45–2.64	3.84–5.04
3.	Annual Mean Temperature (^0^C)	6.9–11.1	15.4–19.6	≥23.8
4.	Soil pH at 5-15cm soil depth	4.7–5.4	6.1–6.6	6.5–7.4
5.	Exchangeable calcium at 20–50cm (cmolc/kg)	≤2.53	8.39–14.25	20.11–25.97

Highest probability (0.695–0.927) cover most optimal values for environmental variables predicting suitable niche that supports survival and distribution of anthrax spores while low probability (≤0.0001) cover less optimal values

The restricted suitable niche favoring survival of anthrax spores predicted in Fig 5 is defined by drier parts of QEPA receiving the least amount of annual precipitation; and bearing the highest levels of exchangeable soil potassium; high annual mean temperatures; alkaline soil pH rich in exchangeable Calcium ions (S3 & S4 Figs). Levels of predicted suitable calcium ions were however distributed both within and beyond the restricted range of suitable habitat. Distribution of suitable levels of potassium was mostly restricted along water shorelines and matched areas with high occurrence of anthrax cases. Parts of the study area receiving high precipitation (1,264–1,394mm annual rainfall); and lower levels of temperatures <15.3°C, pH <5.0, fell within areas predicted as least suitable niche for anthrax occurrence.

## Discussions

The predicted suitable niche in this study was defined by a narrow-restricted corridor bearing hot-dry climatic conditions with alkaline soils rich in potassium and calcium ions. Despite the historical presence of anthrax in QEPA, and its associated impacts on wildlife and public health [18, 30, 31], ecological drivers of anthrax in the ecosystem have not been assessed. This study presents the first estimation of the geographic potential for suitable landscape and environmental conditions that have the potential to support survival and distribution of *B*. *anthracis* spores at the study area. Given the location of QEPA astride the equator, and on the floor of a Rift Valley, the predicted hot-dry climatic conditions and soil properties would be expected to affect the study area uniformly, but this was not the case. The high-risk locations were defined by a narrow-restricted corridor bearing a north-east to south-westward direction spanning from just above Lake George downwards to L. Edward including shorelines of water bodies (Fig 5).

Hippos are the most susceptible wildlife species that suffer significant mortality due to anthrax at the study area [30], and contributed over 80% of the occurrence localities used for the ENM modelling in this study. Being semi-aquatic animals, hippos live in water but graze on land. They have a heavily grazed but highly restricted grazing range spanning 3–6 km from water shorelines where they dwel [44], which is where they presumably get exposed to anthrax spores. However, most return to die in water and their carcasses are found on shorelines of the inhabited waterbodies [18, 30]. This was thought to be a potential notable limitation for this study, since it would seem as though the predicted suitable niche along shorelines of waterbodies simply reflects areas where hippo anthrax carcasses occurred rather than hotspots for anthrax persistence. However, results from an independent model built using buffalo-only occurrence localities was not significantly different (S2 Fig) from the model built using all cases including hippos (Fig 5). The predicted suitable anthrax niche featuring a narrow southwest-northeast corridor spanning from north of L. Edward to north of L. George and the associated shorelines remained outstanding potential hot zones for persistence of anthrax in the study ecosystem. Thus, the potential of hippos in supporting the animal-soil-animal infectious cycle of *B*. *anthracis* [3] remains crucial, as this corridor is co-located with hippo habitat, and is clearly critical for spore transmission and survival steps. Ultimately, the narrow belt predicted for anthrax persistence was also defined by the most optimal levels of the five most important environmental covariates that significantly contributed to the predictive power of the model. Thus, the belt defined the drier parts of the park receiving the least amount of annual precipitation (≤852mm); and bearing the highest levels of: - 1) exchangeable potassium (3.84–5.04 cmolc/kg); 2) annual mean temperatures (23.8°C); 3) soil pH (6.5–7.4); and 4) exchangeable calcium (20.11–25.97cmolc/kg) (Table 5). This suggests the significance of these variables in facilitating anthrax persistence at the study environment.

However, some authors prefer using only laboratory positive occurrence data for ENM modelling to increase the power of the model in accurately predicting the suitable ecological niche [24]. In this study, we used both clinical and laboratory confirmed occurrence data but addressed this limitation by rigorously screening case data [30] using a standard practice for epidemiological investigations by defining anthrax outbreak cases based on pathognomonic clinical, postmortem and or laboratory diagnostic criteria [2, 45]. Given that the predicted suitable niche and hot-dry climatic conditions found in this study agree well with historical anthrax outbreak patterns demonstrated in earlier studies [18, 31], we believe any of these potential limitations did not significantly affect the study findings.

The predicted low rainfall belt was particularly very distinct (S3 Fig), with a significantly high suitability score (0.90) for the annual precipitation variable (Table 4), implying this variable was critical in providing predictive power of the model for detecting anthrax presence. This finding is in agreement with the low rainfall belts defined in the map of Isohyets of QEPA in 1964–1966 [28]. Isohyets are meteorological lines drawn on a map that connect different geographical locations receiving similar amounts of rainfall [46]. The suitable niche for anthrax occurrence in this study area is found within Field’s isohyet lines receiving mean annual precipitation of <1,000 mm equivalent [28]. The hot-dry climatic conditions found to predict anthrax risk in this study have long been reported in anthrax outbreaks in other ecoregions as factors facilitating spore survival or precipitating anthrax outbreaks [2, 7, 14, 25]. Severe droughts or dry seasons preceded or followed by heavy rains, and severe seasonal variations in rainfall patterns [2, 47, 48] are typical events reported to elicit outbreaks. For the susceptible hosts, these climatic conditions also result in nutritional deficiencies that impair host resistance and increase susceptibility [2, 14]. These findings are also in agreement with patterns of major historical anthrax outbreaks that have been recorded at the study area between 1964–2011 [18, 30, 31], which indicate that outbreaks usually started with the onset of rains following prolonged dry spells or with the onset of the dry season following rains. First rains following dry spells are important for the dispersal of spores in runoff water to low lying areas believed to be spore concentration points. These concentration points then become sources of primary index case outbreaks under conducive environmental conditions [3, 14, 47].

The observed restricted niche in this study is similar to that reported by Blackburn *et al* in ecological niche models and other similar studies for anthrax distribution in the United States [9, 47]. Their study attributed the range restriction for anthrax persistence to distribution of *B*. *anthracis* spores along cattle movement trails, trade routes and industrial areas for processing cattle products. In fact, cattle movement corridors are historical routes that have long been associated with the spread of anthrax [2]. In Uganda, historical livestock anthrax outbreaks occurred in cattle producing provinces of the country (northern, eastern and western), along cattle corridors, trade cattle quarantine areas, and within cattle consuming regions of the country [31]. The accounts of S.G. Wilson in 1947, and J.I. Taylor in 1950 [31] illustrate the severity of historical livestock outbreaks along cattle trade routes then, and how vaccination of trade cattle controlled the outbreaks. QEPA falls on the South-western outskirts of the major national cattle corridor [48] and within a high-density cattle region. Most of the key wildlife species in the park do not have a specific migratory pattern or corridor that would be expected to influence anthrax spore distribution. However, the *Hippopotamus amphibious* has a heavily grazed restricted terrestrial grazing range spanning 3–6 km from water shorelines where they dwell [44]. Critically, this range fell within the predicted suitable anthrax niche. Given the high susceptibility of hippos to anthrax, and the historically high mortalities suffered, their population densities, feeding and social behaviors have been hypothesized to enhance spread, propagation, and sustenance of anthrax spores at the study area [30]. The current study confirms this risk, given the high overlap between hippo habitat, hippo feeding areas and the identified high-risk habitat for anthrax survival and exposure.

Haemorrhagic fluids and body exudates from dead infected hosts seed soils with anthrax spores [5]. This is an important step for establishing the animal-soil-animal infectious cycle for *B*. *anthracis* [3], but propagation of the infection relies on the next herbivorous host ingesting or inhaling viable, infective spores [2, 7]. The ingestion of viable and infective spores, in turn, relies on the longevity of spores shed in the soil and their subsequent germination and capacity for infectivity which is in turn influenced by combinations of environmental conditions which define the suitable environment for spore survival [4, 12]. The study of Driciru *et al* [30] analysed two severe anthrax outbreaks in hippos at the study area and observed that cases occured as point-source-propagated outbreaks, suggestive of common source areas where primary cases were exposed to viable, infective spores before propagating the disease. Findings of the current study suggests that the suitable environment for initiating anthrax propagation from these common source areas is dependent primarily on soil pH as well as calcium and potassium ion concentrations, favourable rainfall and ambient temperatures.

Calcium and pH are soil properties well known to support survival and germination of *B*. *anthracis* spores [4, 12]. In this study, high levels of soil calcium and pH showed a significantly high marginal response for the model for prediction of suitable niche for anthrax persistence (Figs 6 & 7), with suitability scores for these corresponding variables going as high as 0.91 and 0.90 respectively (Table 4). The spatial distribution of suitable pH levels showed an outstanding match to the predicted niche (S3 Fig), as well as that of calcium (S4 Fig) which was more widely spread. This is expected since location of the study area is within a Rift Valley system with geological properties rich in gypsum-rich calcareous soils [27, 28], reported to be prone to anthrax outbreaks [2, 3, 14]. The study findings that identify pH and calcium as suitable variables for predicting anthrax occurrence is comparable to studies of Dragon *et al* [2, 4, 14, 49, 50] that demostrate the importance of pH and calcium in spore biology. Bacterial spores contain a significant amount of calcium, which plays an essential role in spore preservation, viability, and germination [12]. Earlier studies have shown that spores of *Bacillus species* are enriched with metallic ions [4]. Uptake of the cations occurs during the sporulation process, and 95% of calcium ions taken gets deposited in the core region of the spore, where it combines with dipicolinic acid to form a salt lattice that stabilizes the DNA and enzymes in the core [4, 12]. This process is believed to increase the thermo resistance properties, and resilience of spores during periods of dormancy [51] and greatly accounts for spore longevity and viability in the environment [4, 12]. Absorbed calcium deposited in spore integument plays an important role in spore germination processes [4, 12], in a manner that is reported to be influenced by soil pH, temperature, water activity and cation levels; relative humidity and seasonal climatic factors [2, 52].

In this study, exchangeable potassium ions featured as the second most important variable (suitability score, 0.95) that contributed to the development and predictive power of the model in determining anthrax presence (Table 4). Increasing levels of potassium, greatly improved performance of the model in predicting presence of anthrax (Fig 6). Distribution of predicted optimal levels of potassium ions along shorelines of water bodies was particularly conspicuous and overlaps areas where most anthrax carcasses were registered and or point source outbreaks postulated to originate from in an earlier study [30]. Information on the significance of potassium in spore biology, infectious or environmental cycles of anthrax is not widely reported, but uptake of different types of metal cations by spores during sporulation is reported to be non-preferential and the metal content of a spore is said to be influenced by the relative concentrations of metals in the sporulation environment [12]. However, once formed, the spore integument reportedly has a definite affinity for certain types of ions [12], and the required levels of preferred ions in the spore are then achieved through a cation exchange process where sodium and magnesium ions for instance eventually get exchanged for the required calcium [12, 53].

Several ecological niche studies conducted at larger spatial resolution identify soil types as important environmental variables influencing distribution and survival of anthrax spores [24], but in this study, soil properties and their mineral elements had more significant influence than soil types per se. This could be attributed to the small study extent and high spatial resolution (1 km grid cells) used, where all soil types within the predicted niche uniformly fell within Harrop’s classification of “soils derived from volcanic rocks, types 5 & 6” and “volcanic and Pre-Cambrian rocks”, mostly comprising black sandy clay loams or clay looms [54, 26].

## Conclusion and recommendations

The predicted hot-dry climatic conditions with alkaline soils rich in potassium and calcium ions found in this study suggest presence of key ecological drivers well known to facilitate survival of *B*. *anthracis* spores and elicit subsequent anthrax outbreaks. The significant associations identified between soil pH, calcium and potassium ion concentrations; and areas identified in earlier studies as potential common source areas where primary hippo cases get exposed to viable, infective spores before propagating the disease, suggests the active role these variabes may play as environmental determinants that actively support anthrax spore survival as well as initiation of outbreaks in the study secosystem. Whether or not favourable levels of these predicted suitable environmental variables experience seasonal variations at the study area remains a subject for longitudinal studies that can improve understanding of ecological drivers of anthrax in the ecosystem.

The fact that the predicted soil properties might originate from geological formations of sedimentary calcareous gypsum rocks has implications for long-term presence of anthrax spores in the park and may explain the long history of anthrax experienced in the area. Identification of suitable niche as a restricted hot zone bounded between low rainfall belts is an important finding that offers opportunities for targeted anthrax surveillance, response and monitoring systems that can aid control and prevention strategies for protecting susceptible wildlife species.

## Supporting information

S1 Fig A sample of historical data archives mined for compiling occurrence records information. Source: Archives from Queen Elizabeth National Park Management Reports for 1956, Kasese, Uganda. The report contains time, location, animal species, case number and diagnostic information required. Location identifiers are in subsequent communication trails.
(TIF)

S2 Fig Suitable niche for anthrax distribution predicted using buffalo cases. This model aimed at assessing if predicted hotspots were majorly influenced by hippo cases as a confounding factor. The suitability map did not show a significantly different outcome.
(TIF)

S3 Fig Distribution of varying levels of annual precipitation and soil pH within predicted suitable ecological niche for *B*. *anthracis* occurrence in QEPA. The precipitation map was built using bioclimatic raster data files obtained from the WorldClim online resources (http://worldclim.org/version2), and published with the kind permission of Dr. Stephen Fick, geo-spatial data scientist [20]. Green colour represents the lowest precipitation level, but most suitable environment for anthrax spore survival, and red represents the highest precipitation and least suitable areas for anthrax distribution.
(TIF)

S4 Fig Distribution of varying levels of exchangeable soil calcium and potassium within predicted suitable ecological niche for *B*. *anthracis* occurrence in QEPA. Maps for soil pH, exchangeable potassium and calcium were built using raster data files obtained from ISRIC online database for Africa SoilGrids resources (https://www.isric.org/projects/soil-property-maps-africa-20-m-resolution), and published with the kind permission of Niels Batjes, Senior Soil Scientist and Coordinator of the World Data center for soils at ISRIC–World Soil Information.
(TIF)

S1 Table Table containing correlation data used for screening predictor variables and response variable data used for modeling. (XLSX)
