# Supplementary figures and images for: Environmental determinants influencing anthrax distribution in Queen Elizabeth Protected Area, Western Uganda
Source: PLoS One. 2020 Aug 18;15(8):e0237223. doi: 10.1371/journal.pone.0237223 (PMC7446795; doi:10.1371/journal.pone.0237223)

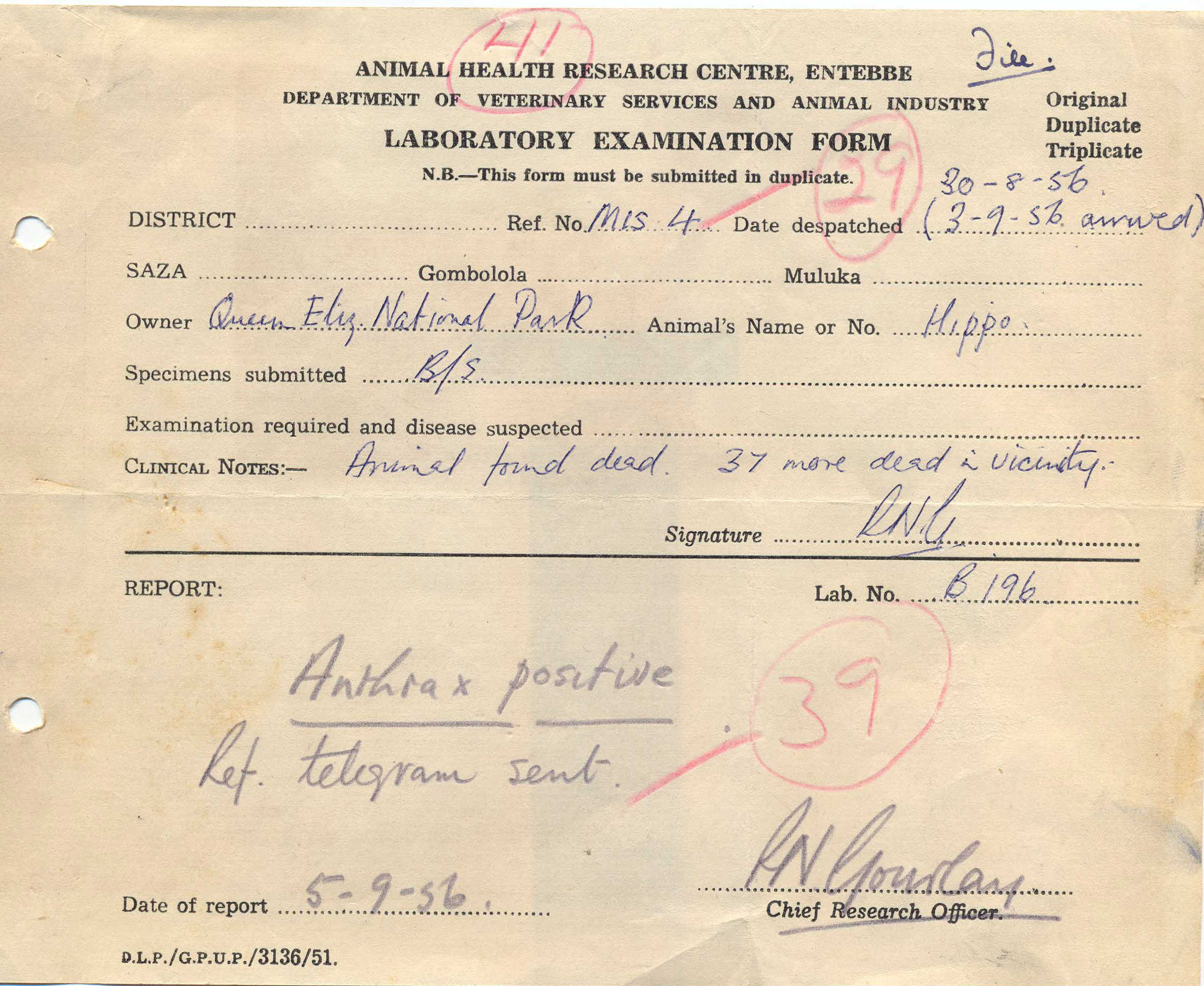

Supplement: S1 Fig — Source: Archives from Queen Elizabeth National Park Management Reports for 1956, Kasese, Uganda. The report contains time, location, animal species, case number and diagnostic information required. Location identifiers are in subsequent communication trails. (TIF) [file pone.0237223.s001.tif]

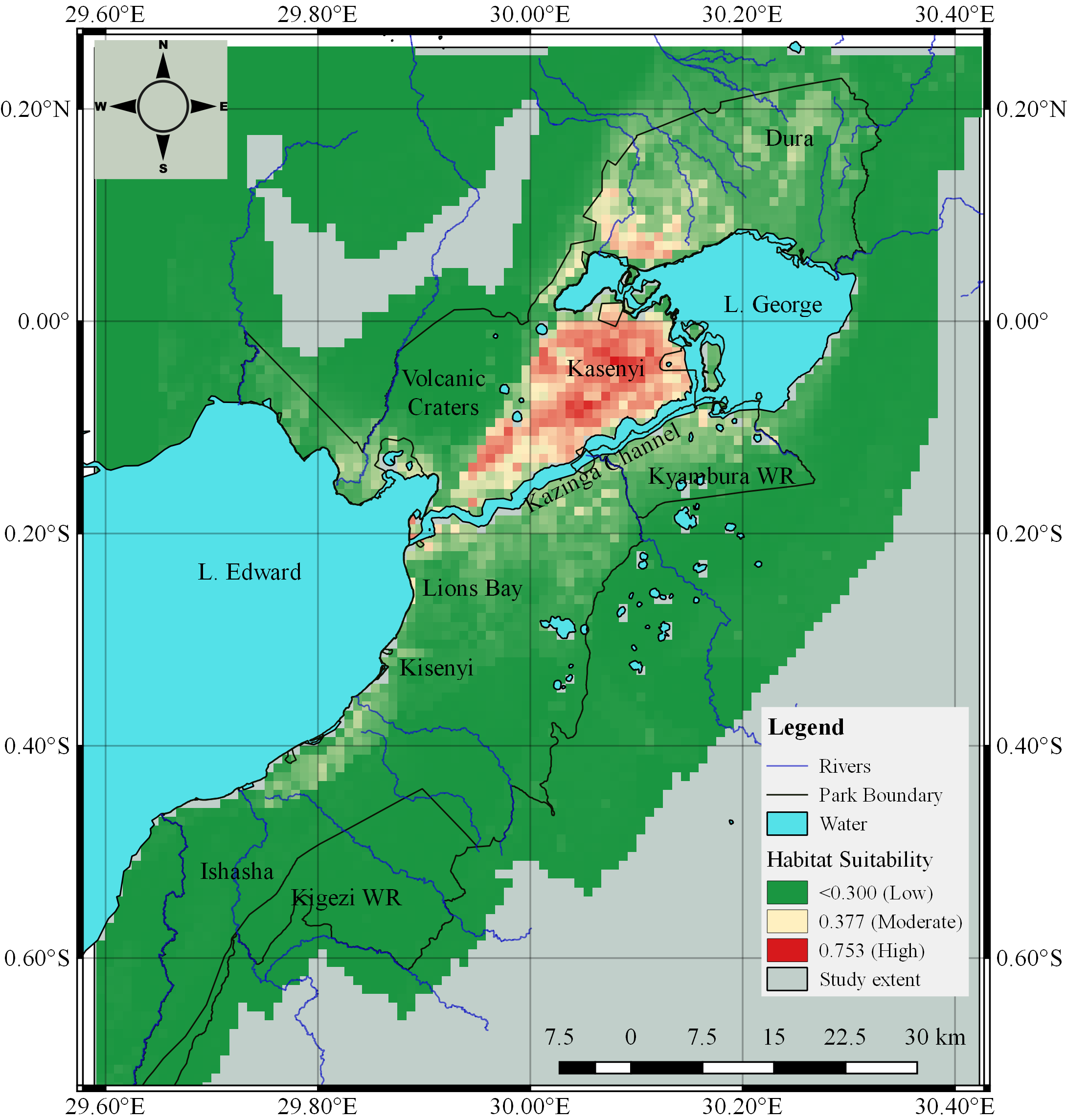

Supplement: S2 Fig — This model aimed at assessing if predicted hotspots were majorly influenced by hippo cases as a confounding factor. The suitability map did not show a significantly different outcome. (TIF) [file pone.0237223.s002.tif]

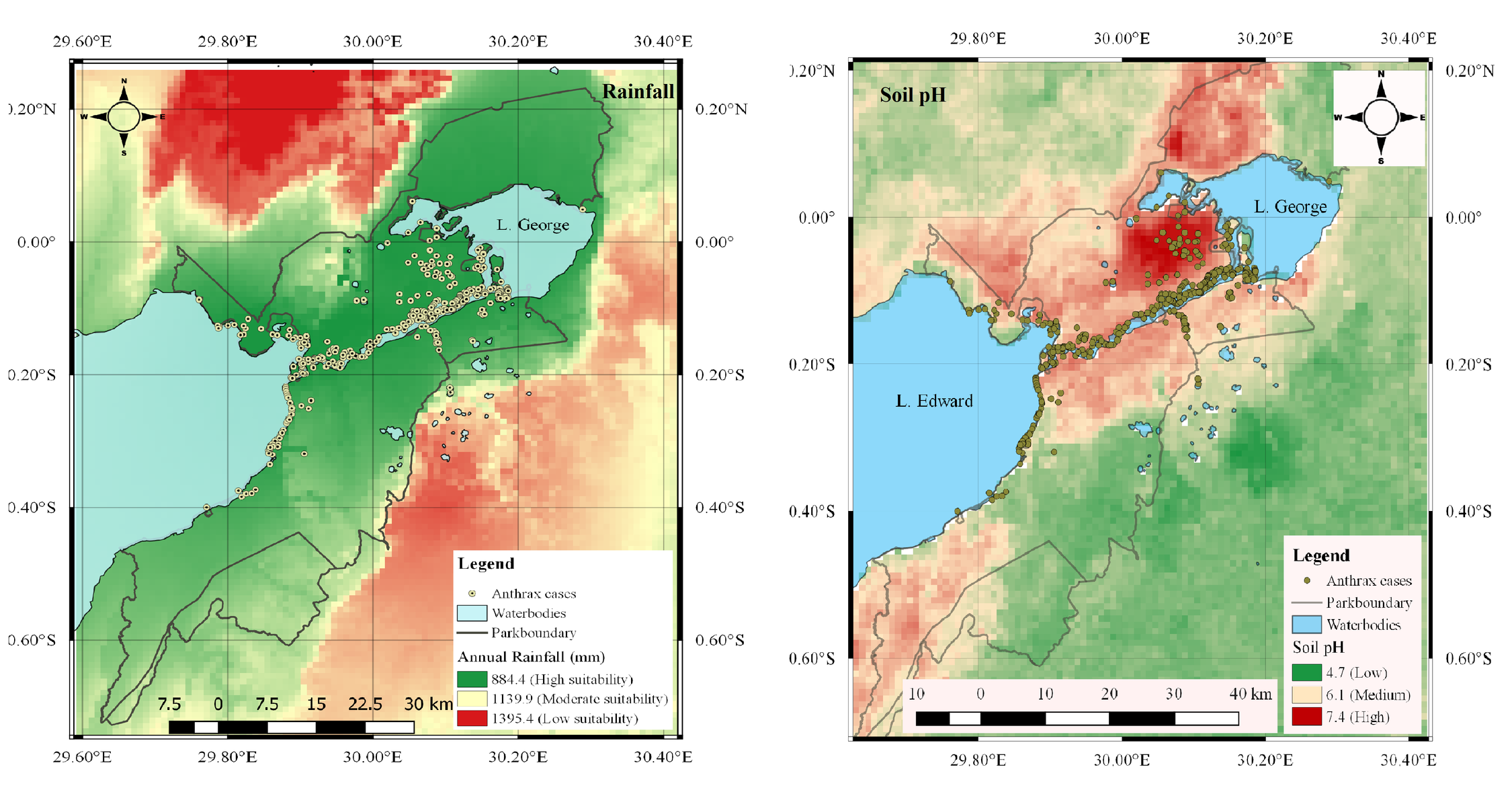

Supplement: S3 Fig — The precipitation map was built using bioclimatic raster data files obtained from the WorldClim online resources (http://worldclim.org/version2), and published with the kind permission of Dr. Stephen Fick, geo-spatial data scientist [20]. Green colour represents the lowest precipitation level, but most suitable environment for anthrax spore survival, and red represents the highest precipitation and least suitable areas for anthrax distribution. (TIF) [file pone.0237223.s003.tif]

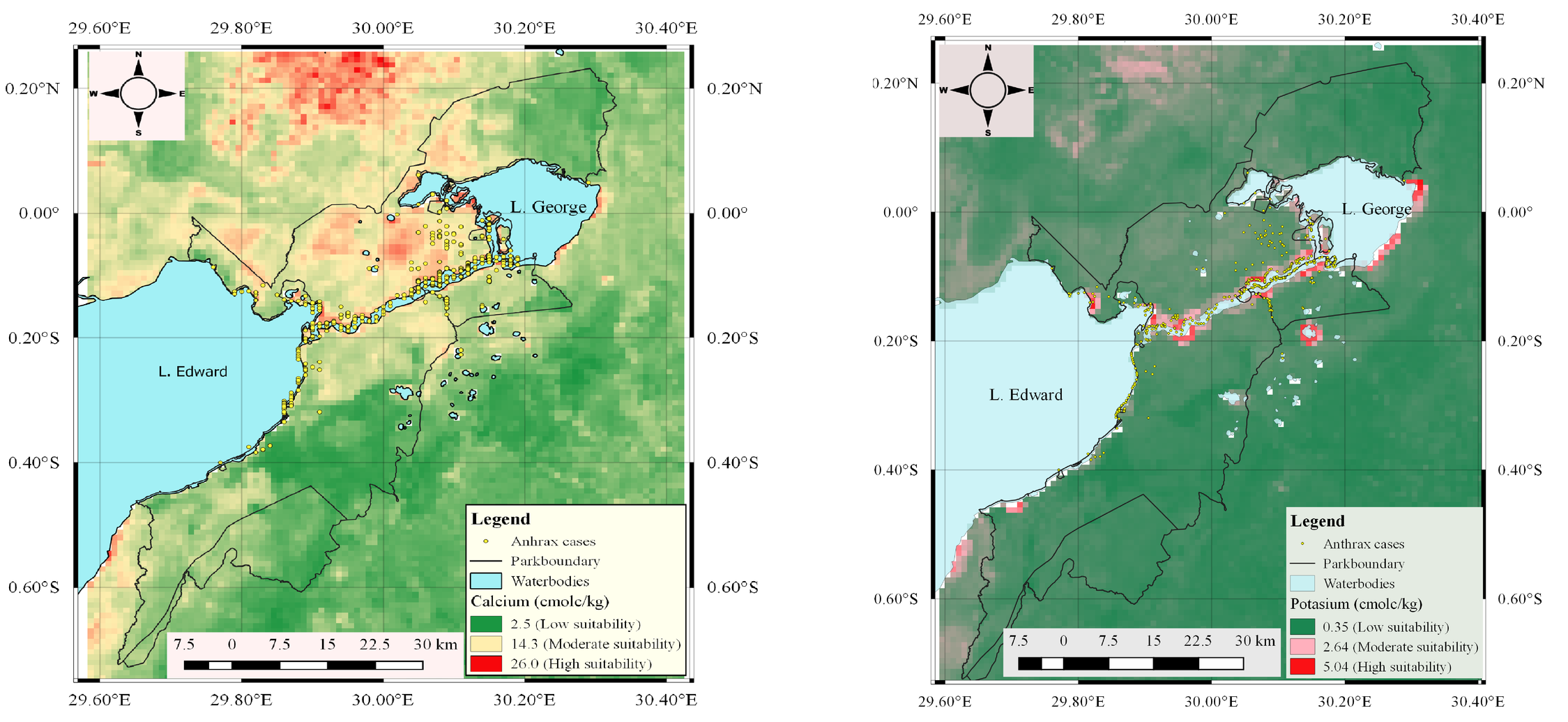

Supplement: S4 Fig — Maps for soil pH, exchangeable potassium and calcium were built using raster data files obtained from ISRIC online database for Africa SoilGrids resources (https://www.isric.org/projects/soil-property-maps-africa-20-m-resolution), and published with the kind permission of Niels Batjes, Senior Soil Scientist and Coordinator of the World Data center for soils at ISRIC–World Soil Information. (TIF) [file pone.0237223.s004.tif]
